# Lithium’s effects on serum neurofilament light in Parkinson’s disease: A post hoc analysis

**DOI:** 10.1016/j.ibneur.2026.03.003

**Published:** 2026-03-14

**Authors:** Thomas Guttuso, Rachel Shepherd, Daniel Sirica, Gregory E. Wilding

**Affiliations:** a Department of Neurology, Jacobs School of Medicine and Biomedical Sciences, University at Buffalo, 5851 Main Street, Williamsville, NY 14221, United States; b Department of Biostatistics, School of Public Health and Health Professions, University at Buffalo, 719 Kimball Hall, Buffalo, NY 14214, United States

**Keywords:** Lithium, Parkinson’s disease, Neuroprotection, Disease-modification, Clinical trial, Neurofilament light

## Abstract

**Background:**

Serum neurofilament light chain (NfL) reflects neuronal degeneration and is likely a disease-progression biomarker in Parkinson’s disease (PD). Therapies shown to decrease serum NfL in PD may provide disease-modifying effects. Serum glial fibrillary acidic protein (GFAP) may predict more rapid cognitive decline in PD.

**Methods:**

Serum samples from two PD trials were assessed for NfL and GFAP using the SIMOA platform at baseline and after 24-weeks of lithium therapy. Post hoc, patients were divided into three groups defined by their serum lithium levels at week 24: “high lithium” (0.21–0.56 mmol/L, median=0.32 mmol/L, *n* = 10), “medium lithium” (0.14–0.20 mmol/L, median=0.17 mmol/L, *n* = 8) and “low lithium” (<0.10–0.12 mmol/L, median<0.10 mmol/L, *n* = 10). Pairwise comparisons were performed using the Fisher-Pitman permutation test.

**Results:**

Median % 24-week changes in serum NfL were −12.8, −2.0 and 11.2 in the high, medium and low lithium groups, respectively. Pairwise group comparisons showed significant differences between high and low lithium (*p* = 0.0001) and high and medium lithium (*p* = 0.0203) but not medium and low lithium groups (*p* = 0.0907). Median % 24-week changes in serum GFAP were 7.3, 42.8 and 12.4, respectively. Pairwise comparisons showed a significant difference between the high and medium lithium (*p* = 0.0075) but not the high and low lithium (*p* = 0.1763) or medium and low lithium groups (*p* = 0.3950).

**Conclusion:**

In this post hoc analysis from two small clinical trials, lithium aspartate therapy achieving a median serum lithium level of 0.32 mmol/L was associated with a significant reduction in serum NfL in PD. Because serum NfL is likely a disease-progression biomarker in PD, further clinical investigation of lithium’s effects on serum NfL and potential disease-modifying effects in PD is merited.

**Conclusion:**

“High lithium” therapy, achieving a median serum level of 0.32 mmol/L, was associated with a significant reduction in serum NfL in PD. Because serum NfL is likely a disease-progression biomarker in PD, further research is merited on lithium’s potential disease-modifying effects in PD

## Highlights

• Serum neurofilament light chain (sNfL) likely reflects neurodegeneration in PD.
• Lithium therapy was associated with a significant reduction in sNfL in PD.
• A median serum lithium level of 0.32 mmol/L showed the largest reductions in sNfL.
• Lithium is the first therapy associated with a significant reduction in sNfL in PD.
• Further research on lithium’s potential disease-modifying effects in PD is merited.

## Graphical Abstract

**Figure:**
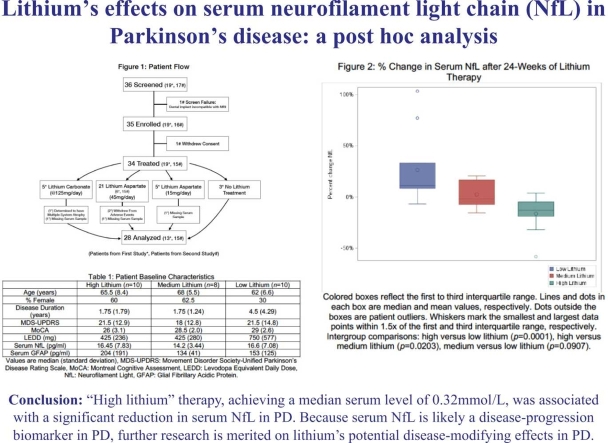

## 1 Introduction

Neurofilament light chain (NfL) is a protein expressed exclusively in neurons and is released into the extracellular space by processes causing neuronal damage or death (Khalil et al., 2018). In Parkinson’s disease (PD), blood NfL levels are elevated at baseline and more rapidly increase longitudinally compared to healthy controls. Blood NfL levels also predict and longitudinally correlate with worsening motor and cognitive symptoms (Mollenhauer et al., 2020, Pedersen et al., 2024, Niemann et al., 2021, Pilotto et al., 2024, Ygland Rodstrom et al., 2022, Lin et al., 2019). As a result, blood NfL is likely a disease-progression biomarker in PD that may be able to reflect an experimental therapy’s disease-modifying benefit (Mollenhauer et al., 2020). Although no therapy has been shown to reduce blood NfL in PD, virtually all of the FDA-approved disease-modifying therapies for multiple sclerosis (MS) significantly reduce blood NfL with the magnitude of clinical disease modification and NfL reduction being significantly correlated (Delcoigne et al., 2020). Reduction of plasma NfL was also a critical factor resulting in FDA approval of tofersen as a disease-modifying therapy for SOD1-amyotrophic lateral sclerosis (ALS) (NeurologyLive, 2025). Although MS, ALS and PD have distinct pathophysiology, because they all share blood NfL as a disease-progression biomarker, therapies shown to significantly reduce blood NfL in PD would suggest disease-modifying benefits worthy of further clinical investigation.

Lithium has multiple neuroprotective actions including suppressing microglial activation, reducing inflammation and oxidative stress, and enhancing autophagy and mitochondrial biogenesis and function (Arraf et al., 2012, Dong et al., 2014, Hou et al., 2015, Struewing et al., 2007, Sarkar et al., 2005). Lithium treatment has demonstrated benefit in several PD animal models (Lieu et al., 2014, Youdim and Arraf, 2004, Zhao et al., 2019, Kim et al., 2011). There is also supportive, albeit tangential, epidemiologic evidence of lithium’s neuroprotective effects based on the 77% reduced risk of PD in smokers shown in prospective cohort studies and the high levels of lithium in tobacco (Guttuso et al., 2019, Ritz et al., 2007, Jathar et al., 1980).

Based on this background, our group performed a pilot clinical trial randomizing 16 PD patients to lithium carbonate (@125 mg/day titrated to achieve a serum lithium level of 0.4–0.5 mmol/L), lithium aspartate 45 mg/day or lithium aspartate 15 mg/day for 24 weeks assessing changes in blood-based and MRI biomarkers; an additional three PD patients served as controls and did not receive lithium (Guttuso et al., 2023). The primary MRI biomarker outcome was free water, which reflects neuronal degeneration and inflammation as well as longitudinal changes in motor and cognitive symptoms in PD (Burciu et al., 2017, Guttuso et al., 2022, Pasternak et al., 2012). Results from the pilot study showed 24 weeks of lithium aspartate therapy 45 mg/day to be associated with the largest and most consistent improvements in the assessed biomarkers compared to the other two lithium dosages; however, only four patients received this dosage and only two of these four patients received MRIs due to funding constraints (Guttuso et al., 2023). As a result, another trial was needed to further explore lithium’s effects on these biomarkers as well as serum NfL. Because two of the six patients who initiated lithium aspartate at a dosage of 45 mg/day withdrew from the pilot study due to side effects, the follow-up trial utilized a lithium aspartate dose titration schedule to hopefully improve tolerability.

Here we report on the combined serum NfL results from the 13 patients from the pilot study reviewed above for whom serum was available and the 15 patients from the trial presented below. Serum glial fibrillary acid protein (GFAP) was also assessed as it was included by the vendor for no additional cost and may reflect and predict cognitive impairment in PD (Tang et al., 2023). We chose to study lithium aspartate over the other available lithium dietary supplement, lithium orotate, due to several animal studies showing orotate use to be associated with higher rates of several cancers, (Columbano et al., 1982, Kokkinakis and Albores-Saavedra, 1994, Kokkinakis et al., 1991) a finding never associated with aspartate use. We chose to study lithium aspartate instead of prescription lithium carbonate due to the results of the above pilot trial and to enhance patient accessibility to lithium therapy.

## 2 Material and methods

Eligible patients were 45–80 years old; diagnosed with PD by UK Brain Bank Criteria for < 4 years (by TG); had stable PD or psychiatric medication dosages for > 30 or > 60 days, respectively; had no history of brain surgery, stroke, or use of lithium or antipsychotic medication and a Montreal Cognitive Assessment score > 20 at screening (ClinicalTrials.gov ID: NCT06099886). Our previous pilot study had the same patient eligibility criteria with the exception that PD patients of any disease duration were eligible (Guttuso et al., 2023).

At the baseline (BL) visit, patients fasted for 10–12 h before providing a venous blood sample. Serum was stored at -70C until NfL and GFAP were assessed in duplicate by *Quanterix* (Billerica, MA) using the SIMOA platform. Safety laboratory tests included a complete blood count and comprehensive metabolic panel including serum calcium and thyroid stimulation hormone levels (*Kaleida Health Laboratories,* Williamsville, NY). Because lithium aspartate 45 mg/day was the most promising dosage in the pilot study, all 15 patients were placed on lithium aspartate 5 mg capsules starting at 10 mg, 2x/day with the dosage increased by 10 mg/day every week up to 45 mg/day (20 mg qAM and 25 mg qhs) for the remainder of the 24-week treatment period. This lithium titration was used to maximize tolerability. At the 24-week visit, another fasting blood sample was obtained and processed the same as for the BL visit and serum lithium was assessed 10–12 h after the last lithium dose.

After reviewing the serum NfL results, the 15 patients who completed the current study and 13 PD patients from the previous pilot study for whom serum was available were divided into three groups based on serum lithium levels at the 24-week visit: “high lithium” (0.21–0.56 mmol/L, median=0.32 mmol/L, *n* = 10), “medium lithium” (0.14–0.20 mmol/L, median=0.17 mmol/L, *n* = 8) and “low lithium” (<0.10–0.12 mmol/L, median<0.10 mmol/L, *n* = 10). Post hoc pairwise comparisons were performed using the Fisher-Pitman permutation test.

## 3 Theory

A therapy shown to reduce serum NfL in PD would suggest disease-modifying effects worthy of further clinical investigation.

## 4 Results

The study received approval from the University at Buffalo’s Institutional Review Board on May 6, 2023. Seventeen patients provided written informed consent from October 2023-June 2024. One patient withdrew prior to starting lithium therapy and one failed screening. Four patients receiving lithium reported mild adverse events of sedation, nausea or dyskinesia that did not require lithium dosage reduction. There were no clinically meaningful changes in any safety laboratory values.

**Fig. 1:**
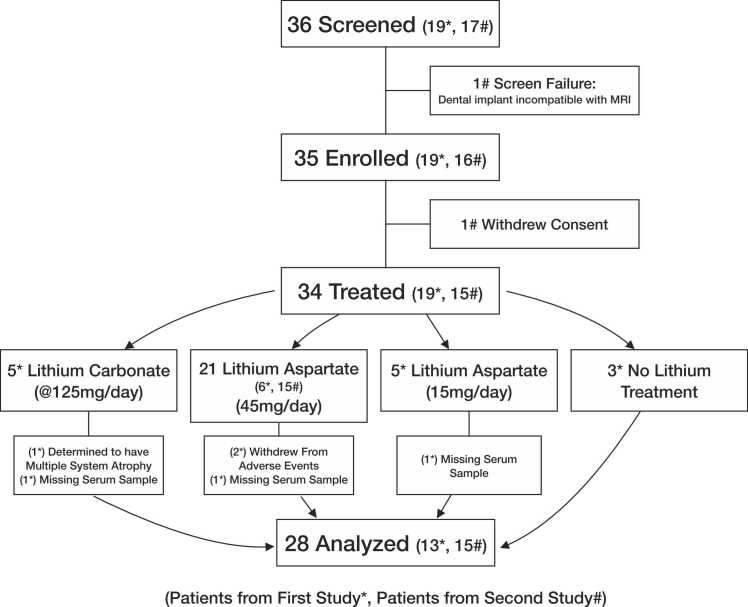
Patient Flow

Patient flow and baseline characteristics for all 28 analyzed patients from both studies are summarized in Fig. 1 and Table 1, respectively. Patients in the low lithium group were slightly younger, had fewer females, longer disease duration and higher levodopa equivalent daily dose compared to the other two groups (Table 1).

**Table 1:** Patient Baseline Characteristics.

	High Lithium (*n* = 10)	Medium Lithium (*n* = 8)	Low Lithium (*n* = 10)
Age (years)	65.5 (8.4)	68 (5.5)	62 (6.6)
% Female	60	62.5	30
Disease Duration (years)	1.75 (1.79)	1.75 (1.24)	4.5 (4.29)
MDS-UPDRS	21.5 (12.9)	18 (12.8)	21.5 (14.8)
MoCA	26 (3.1)	28.5 (2.0)	29 (2.6)
LEDD (mg)	425 (236)	425 (280)	750 (577)
Serum NfL (pg/ml)	16.45 (7.83)	14.2 (3.44)	16.6 (7.08)
Serum GFAP (pg/ml)	204 (191)	134 (41)	153 (125)

Values are median (standard deviation), MDS-UPDRS: Movement Disorder Society-Unified Parkinson’s Disease Rating Scale, MoCA: Montreal Cognitive Assessment, LEDD: Levodopa Equivalent Daily Dose, NfL: Neurofilament Light, GFAP: Glial Fibrillary Acidic Protein.

**Fig. 2:**
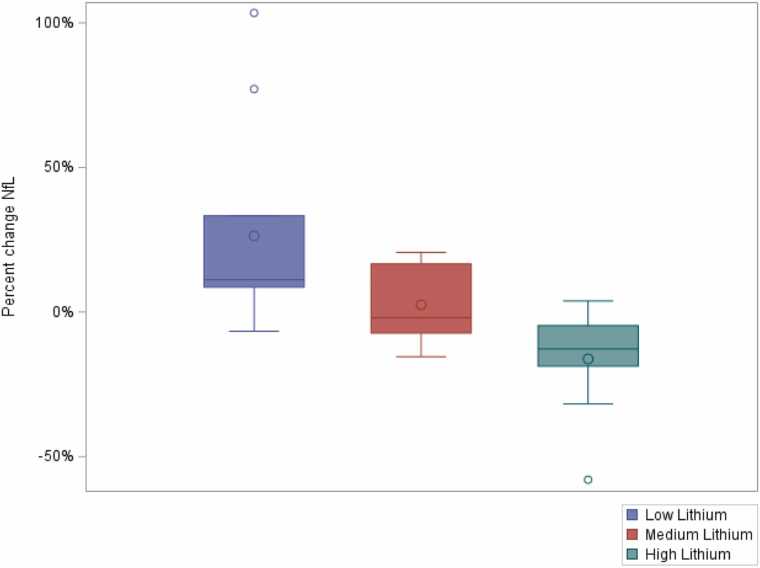
% Change in Serum NfL after 24-Weeks of Lithium Therapy. Colored boxes reflect the first to third interquartile range. Lines and dots in each box are median and mean values, respectively. Dots outside the boxes are patient outliers. Whiskers mark the smallest and largest data points within 1.5x of the first and third interquartile range, respectively. Intergroup comparisons: high versus low lithium (*p* = 0.0001), high versus medium lithium (*p* = 0.0203), medium versus low lithium (*p* = 0.0907).

Median % changes in serum NfL were −12.8, −2.0 and 11.2 in the high, medium and low lithium groups, respectively (Fig. 2). Pairwise comparisons showed significant differences between the high and low lithium groups (*p* = 0.0001) and high and medium lithium groups (*p* = 0.0203) but not the medium and low lithium groups (*p* = 0.0907).

**Fig. 3:**
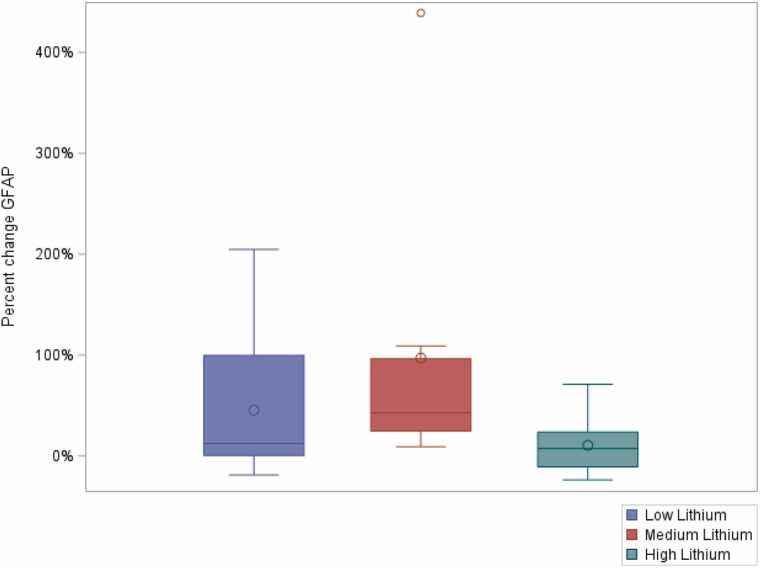
% Change in Serum GFAP after 24-Weeks of Lithium Therapy. Colored boxes reflect the first to third interquartile range. Lines and dots in each box are median and mean values, respectively. Dots outside the boxes are patient outliers. Whiskers mark the smallest and largest data points within 1.5x of the first and third interquartile range, respectively. Intergroup comparisons: high versus low lithium (*p* = 0.1763), high versus medium lithium (*p* = 0.0075), medium versus low lithium (*p* = 0.3950).

Median % changes in serum GFAP were 7.3, 42.8 and 12.4 in the high, medium and low lithium groups, respectively (Fig. 3). Pairwise comparisons showed a significant difference between the high and medium lithium groups (*p* = 0.0075) but not the high and low lithium groups (*p* = 0.1763) or the medium and low lithium groups (*p* = 0.3950).

## 5 Discussion

To our knowledge, this is the first report of any therapy being associated with significant reductions in serum NfL in PD and the first report of lithium therapy having this effect in any patient population. Although the results are only based on 28 patients from two separate studies and should be considered preliminary, they suggest a dose-response relationship between serum lithium levels and changes in serum NfL (Fig. 2). The pairwise comparisons indicate the high lithium group, with serum lithium levels of 0.21–0.56 mmol/L, median 0.32 mmol/L, as being associated with the largest reductions in serum NfL. Although patients in the low lithium group had fewer females, longer disease duration and higher LEDD at baseline compared to the other two groups, there still was a significant difference between the high and medium lithium groups, which had very similar baseline patient characteristics (Table 1). Regarding the low lithium group baseline differences, it is possible that male PD patients of longer disease duration may have relatively greater longitudinal increases in sNfL, which would have contributed to the intergroup differences. However, a previous report showed greater longitudinal sNfL increases in early compared to later PD, (Pedersen et al., 2024) which would predict the low lithium group to have relatively less of a longitudinal increase in sNfL. Intergroup differences in baseline characteristic not assessed could also have influenced the results.

In contrast, there was less of an increase in sGFAP in the high lithium group compared to the medium but not the low lithium group (Fig. 3). It is not clear what may expain the disparate results between the sNfL and sGFAP biomarkers in our analyses; however, another baseline difference in the low lithium group may have contributed. The low lithium group had a relatively higher median MoCA score despite a much longer median disease duration (Table 1) indicating relatively less cognitive impairment. Because sGFAP is elevated in PD with more advanced cognitive impairment and predicts cognitive decline, (Tang et al., 2023) the relatively lower degree of cognitive impairment in the low lithium group may have contiributed to a lower longitudinal increase in sGFAP than if the groups were better matched on this baseline characteristic.

Serum GFAP reflects glial activation and neuroinflammation, but not necessarily neurodegeneration. Serum NfL, on the other hand, specifically reflects neuronal damage and degeneration and, thus, may be a more sensitive biomarker for assessing PD progression as well as the disease-modifying effects of experimental therapeutics (Mollenhauer et al., 2020, Pilotto et al., 2024). Another advantage of serum NfL is that it reflects neurodegeneration from multiple causes (Khalil et al., 2018). While the pathological hallmark of PD is intraneuronal aggregated alpha-synuclein, about 50–79% of PD cases have additional pathologies including aggregated tau, beta amyloid and TDP-43 as well as vascular pathology (Walker and Attems, 2024, Buchman et al., 2021, Dugger et al., 2014, Chu et al., 2024, Zhang et al., 2018). Because combinations of these diverse pathologies likely contribute to neurodegeneration in PD, (Spires-Jones et al., 2017) use of a disease-progression biomarker that reflects neurodegeneration from many pathological causes, such as serum NfL, is arguably preferable for use in identifying disease-modifying therapies than biomarkers specific to any single PD pathology. In addition, serum NfL is currently the only biomarker shown to reflect disease-modifying effects of distinct therapies across multiple neurological diseases, (Delcoigne et al., 2020, NeurologyLive, 2025) as previously noted, supporting its potential to reflect therapeutic disease-modifying effects in other neurological diseases.

Our findings associating significant reductions in serum NfL only in patients with the highest serum lithium levels of 0.21–0.56 mmol/L are consistent with the history of lithium’s clinical use for treating bipolar disorder. In bipolar disorder, lithium’s only FDA-approved indication, its efficacy is dependent on achieving a target serum lithium level of 0.6–0.8 mmol/L in younger patients and 0.4–0.8 mmol/L in older patients (Malhi et al., 2016, Shulman et al., 2019). Serum lithium levels below these ranges are usually clinically ineffective in bipolar disorder. Thus, our biomarker findings of lower serum lithium levels not being associated with significant reductions in serum NfL aligns with this paradigm of lithium’s biological activity being dependent on its serum levels. It should be noted that serum lithium levels > 0.8 mmol/L, particularly in the elderly with bipolar disorder, are more likely to cause clinical side effects as well as toxicities such as chronic kidney disease.

It is reassuring that lithium was well-tolerated in this PD trial, which targeted serum lithium levels about half of those used for treating bipolar disorder. However, because a disease-modifying therapy for PD would likely be initiated early in the disease and continued long-term, there would also need to be reassuring renal safety data on long-term lithium use in older cohorts before it could be considered for use as a possible disease-modifying therapy in PD.

Towards this end, one population-based cohort study found no increased risk of renal dysfunction among 374 older patients (median 69 years old) receiving lithium therapy for a median of 3 years when serum lithium levels were ≤ 0.7 mmol/L (median 0.48 mmol/L); however, there was a significant increased risk of renal dysfunction among 300 patients with serum lithium levels > 0.7 mmol/L (median 0.87 mmol/L) treated for a similar duration (Rej et al., 2020). In addition, a randomized controlled trial in an older cohort of 59 patients (mean 71.6 years old) with mild cognitive impairment showed up to 4 years of “low-dose” lithium therapy (achieving serum levels of 0.25–0.50 mmol/L) to be well-tolerated and not to be associated with any significant changes in renal function (Aprahamian et al., 2014). Considering the average age of PD diagnosis is about 60 years old, these studies support the long-term safety and continued clinical research of lithium therapy in PD targeting serum lithium levels ≤ 0.50 mmol/L.

Limitations of our clinical trial data include its small sample size and lack of patient randomization to the three lithium groups, which may have introduced selection bias, and lack of adjustment for potential confounders. In addition, the lithium groups were defined post hoc, which increases the chance for Type I errors. Finally, the small sample size and short treatment duration in these studies cannot inform how lithium may affect important, long-term PD clinical outcomes like falls, freezing-of-gait and cognitive decline. The main strengths of these data include use of the SIMOA technique for assessing serum NfL, which is considered a gold-standard assay for assessing ultra-low concentration serum proteins, (Pelisek et al., 2024, Truffi et al., 2023) and only including patients with a PD diagnosis confirmed by a movement disorder specialist (TG).

In order to gather pilot data on how higher serum lithium levels influence biomarker changes among the patients from the above studies, we initiated an extension study where patients completing the first 24-week trial are eligible to receive 24-weeks of open-label lithium therapy with the dosage titrated to achieve a target serum lithium level of 0.25–0.50 mmol/L with a goal of 0.45 mmol/L (Clinicaltrials.gov ID: NCT06592014). Anecdotally, we have found serum lithium levels > 0.50 mmol/L to be poorly tolerated in PD, which is why we have set this as the upper limit for the extension study (Guttuso, 2016). Results will increase the power of our analyses and assist in the design of future lithium/PD trials particularly in terms of identifying the most promising target serum lithium level associated with maximum improvements in serum NfL as well as free water assessed by MRI.

## 6 Conclusions

In this pooled, post hoc analysis from two small clinical trials, lithium therapy achieving a median serum level of 0.32 mmol/L was associated with a significant reduction in serum NfL in PD. Considering that blood NfL is likely a disease-progression biomarker in PD and is a validated therapeutic disease-modifying biomarker in MS and ALS, further research is merited investigating lithium’s effects on serum NfL and potential disease-modifying effects in PD.

## CRediT authorship contribution statement

**Guttuso, Jr. Thomas:** Writing – review & editing, Writing – original draft, Supervision, Methodology, Investigation, Funding acquisition, Data curation, Conceptualization. **Daniel Sirica:** Writing – review & editing, Investigation. **Wilding Gregory:** Writing – review & editing, Formal analysis. **Rachel Shepherd:** Writing – review & editing, Investigation, Conceptualization.

## Compliance with Ethical Standards

The authors declare that all experiments on human subjects were conducted in accordance with the Declaration of Helsinki https://www.wma.net/policies-post/wma-declaration-of-helsinki-ethical-principles-for-medical-research-involving-human-subjects/ and that all procedures were carried out with the adequate understanding and written consent of the subjects.

The authors also certify that formal approval to conduct the experiments described has been obtained from the human subjects review board at the University at Buffalo and can be provided upon request.

## Authors’ financial disclosures related to the research reported in the manuscript

Thomas Guttuso, Jr. is president and majority owner of e3 Pharmaceuticals and has received funding from the *Cure Parkinson’s Trust*. None of the other authors have any declarations of interest.

## Data Sharing

All data produced in the present study are available upon reasonable request to the corresponding author.

## Authors’ Roles

Thomas Guttuso, Jr.: conceived the study design and performed most of the study procedures, contributed to data analysis, wrote the original manuscript draft and contributed to manuscript editing with the other authors.

Rachel Shepherd: contributed to the study design, performed some of the study procedures and helped to edit the final manuscript.

Daniel Sirica: contributed to the study design, performed some of the study procedures and helped to edit the final manuscript.

Gregory E. Wilding: performed all statistical analyses, produced the manuscript’s figure and helped to edit the final manuscript.

## Funding

This study was funded by the National Center for Advancing Translational Sciences of the National Institutes of Health (10.13039/100000002NIH) under award number UL1TR001412 to the University at Buffalo. The NIH played no role in the study design; in the collection, analysis and interpretation of data; in the writing of the report; or in the decision to submit the article for publication. Thomas Guttuso, Jr. had full access to all the data in the study and takes responsibility for the integrity of the data and the accuracy of the data analysis.

## Declaration of Competing Interest

Thomas Guttuso, Jr.: President of e3 Pharmaceuticals, Inc. Support from *Cure Parkinson’s Trust*

Rachel Shepherd: None.

Daniel Sirica: None.

Gregory Wilding: None.
